# Mycobacterium Tuberculosis-Specific TNF-α Is a Potential Biomarker for the Rapid Diagnosis of Active Tuberculosis Disease in Chinese Population

**DOI:** 10.1371/journal.pone.0079431

**Published:** 2013-11-11

**Authors:** Feng Wang, Hongyan Hou, Lingqing Xu, Munanie Jane, Jing Peng, Yanjun Lu, Yaowu Zhu, Ziyong Sun

**Affiliations:** Department of Clinical Laboratory, Tongji Hospital, Tongji Medical College, Huazhong University of Science and Technology, Wuhan, China; University of Louisville, United States of America

## Abstract

Interferon-gamma release assays (IGRAs) have proven to be useful to accurately detect *Mycobacterium tuberculosis* (*Mtb*) infection, but they cannot reliably discriminate between active tuberculosis (TB) and latent tuberculosis infection (LTBI). This study aims to test whether *Mtb*-specific tumor necrosis factor-alpha (TNF-α) could be used as a new tool for the rapid diagnosis of active TB disease. The secretion of TNF-α by *Mtb*-specific antigen-stimulated peripheral blood mononuclear cells (PBMCs) of sixty seven participants was investigated in the study. Our results showed that the total measurement of TNF-α secretion by *Mtb*-specific antigen-stimulated PBMCs is not a good biomarker for active TB diagnosis. However, we found that calculation of *Mtb*-specific TNF-α not only distinguish between active and latent TB infection, but also can differentiate active TB from non-TB patients. Using the cutoff value of 136.9 pg/ml for *Mtb*-specific TNF-α, we were able to differentiate active TB from LTBI. Sensitivity and specificity were 72% and 90.91%. These data suggest that *Mtb*-specific TNF-α could be a potential biomarker for the diagnosis of active TB disease.

## Introduction

Tuberculosis (TB) is one of the leading causes of death by infectious diseases worldwide. Approximately one-third of population in the world is infected with *Mycobacterium tuberculosis* (*Mtb*). Of those exposed, eight million develop symptoms and approximately two million die from the infection each year [1]. TB has the special characteristic that most of the infected individuals remain latent. Most individuals with latent TB are asymptomatic for their lifetime, with only approximately 15% ever developing active disease [2]. However, the global problem of TB is worsening with emergence of drug resistant *Mtb* strains and the increased susceptibility of HIV-infected individuals to developing TB [3], [4]. Early diagnosis and treatment of active TB disease are the key points to prevent epidemic of this disease.

There are many methods for diagnosis of *Mtb* infection. Microscopy of acid-fast staining (AFS) and culture for *Mtb* are gold standards for the diagnosis of active TB. However, some patients are unable to produce suitable sputum and culture time is longer than two weeks, leading to unsatisfied demand for clinicians. Although direct smear microscopy is rapid and inexpensive, its sensitivity is limited [5]. The tuberculin skin test (TST) used as a classic way to diagnose active TB disease or latent tuberculosis infection (LTBI) is unreliable, for the reason that it has low sensitivity and specificity for TB infection in clinical use [6]. Molecular approaches such as the Xpert MTB/RIF assay [7], [8] reduce the detection time and have been endorsed by the WHO. Although they are sensitive and relatively easy to use, these tests are costly and cannot completely substitute for classic methods especially in developing countries.

Interferon-gamma release assays (IGRAs) are based on interferon-gamma (IFN-γ) secretion by lymphocytes exposed to *Mtb-*specific antigens such as early secreted antigenic target 6 (ESAT-6) and culture filtrate protein 10 (CFP-10). IGRAs have been shown to be very sensitive and specific for LTBI especially in comparison to the TST [9]–[11]. While IGRAs are useful in the diagnosis of *Mtb* infection, an important limitation of these assays is their inability to discriminate between active TB and LTBI [12]. Thus, IGRAs are of little value in high TB incidence areas with a very high LTBI burden like China.

Discovery of biomarkers that can rapidly distinguish active TB from LTBI would be a major breakthrough in high-TB-prevalence countries. In this study, we demonstrate that *Mtb*-specific tumor necrosis factor-alpha (TNF-α) is a new tool for the rapid diagnosis of active TB disease.

## Materials and Methods

### Study groups

This study was carried out from January to May 2013 at Tongji Hospital (TJH), the largest hospital in central region of China. Participants were selected based on *Mtb*-specific IFN-γ ELISPOT responses routinely performed for the diagnosis of *Mtb* infection at TJH. Subjects were classified into the following four categories: (1) active TB patients; (2) LTBI individuals; (3) non-TB control subjects; (4) healthy volunteer subjects.

Besides positive *Mtb*-specific IFN-γ ELISPOT responses, patients with active TB had a diagnosis based on laboratory isolation of *Mtb* in mycobacterial culture from sputum, broncho alveolar lavage fluid or plerual effusion, and/or AFS, and/or PCR. The final diagnosis was given by a clinician after validation of these criteria associated with clinical symptoms. Individuals who had a positive *Mtb*-specific IFN-γ ELISPOT responses but lacked clinical or radiographic evidence of active TB were diagnosed as LTBI. Patients with clinical symptoms (fever, night sweats, weight-loss) but without evidence of active TB were recruited as non-TB control subjects. Volunteers who had a negative *Mtb*-specific IFN-γ ELISPOT responses and without any pulmonary symptoms or active disease were recruited as healthy control subjects. This study was approved by the ethical committee of Tongji hospital, Tongji Medical College, Huazhong University of Science and Technology, Wuhan, China. All participants gave written consent to the study.

### IFN-γ ELISPOT assay

Peripheral blood mononuclear cells (PBMCs) were isolated from heparinized blood of four groups of participants by using Ficoll-Hypaque density gradients (Sigma-Aldrich, St Louis, MO). *Mtb*-specific IFN-γ ELISPOT assay was performed according to the instruction of T-SPOT.TB kit (Oxford Immunotec, Abingdon, UK).

### ELISA for determination of *Mtb*-specific TNF-α

PBMCs isolated as above were also used for determination of *Mtb*-specific TNF-α. In order to determinate *Mtb*-specific TNF-α, 100 µl of fresh PBMCs (2.5×10^5^/well) were seeded in 96-well plates and were stimulated with 50 µl of ESAT-6, CFP-10 or medium ( ESAT-6 and CFP-10 reagents were the same as T-SPOT.TB kit). The plates were incubated at 37°C for 16–20 h. After incubation, the supernatant was collected after centrifugation. Concentration of TNF-α in culture supernatant was measured by a standard sandwich cytokine ELISA procedure according to the instruction of human TNF-α detection kit (R&D Systems, Minneapolis, MN). *Mtb*-specific TNF-α was defined by the following rule: *Mtb*-specific TNF-α =  TNF-α_ESAT-6 or CFP-10 stimulation_ - TNF-α_background_. TNF-α_background_ is the secretion of TNF-α by medium-stimulated PBMCs.

### Analysis of ESAT-6-induced cytotoxicity

PBMCs were isolated from three non-TB patients. One hundred microliters of PBMCs (2.5 × 10^5^/well) were seeded in 96-well plates and were stimulated with different concentrations (10, 20, 30 µg/ml) of phytohemagglutinin (PHA) (Sigma-Aldrich, St Louis, MO). In some experiments, 50 µl of ESAT-6 was added to culture medium. The plates were incubated at 37°C for 16–20 h. After incubation, the supernatant was collected and concentration of TNF-α in culture supernatant was measured by sandwich ELISA, as above. To further determine ESAT-6-induced cytotoxicity, PBMCs isolated from another five non-TB patients were stimulated with 30 µg/ml PHA for 24 h in the presence or absence of ESAT-6. After culture, the Annexin V-APC Apoptosis Detection Kit (17-8007, eBioscience) was used for detection of apoptosis in PBMCs. All procedures were performed according to the manufacturer’s instructions. Stained cells were then analyzed on FACScalibur using Cell Quest software (Becton Dickinson, San Jose, CA).

### Statistical analysis

The statistical significance of differences in *Mtb*-specific TNF-α concentration among the four groups of participants was evaluated with the Mann-Whitney U test. Differences in percentages of apoptotic cells between nonstimulated and ESAT-6-stimulated PBMCs were assessed using the paired Student’s *t*-test. Receiver operating characteristic (ROC) analysis was performed to determine cutoff levels of *Mtb*-specific TNF-α in discriminating between active TB and LTBI. Statistical significance was determined as *p*< 0.05 (*p< 0.05, **p< 0.001).

## Results

### Participants

Sixty seven participants were included in the study. Ten subjects were classified as non-TB patients, 10 as healthy volunteers, 22 as LTBI individuals and 25 as active TB patients. Active TB disease was diagnosed in 25 subjects on the basis of positive *Mtb*-specific IFN-γ ELISPOT responses, clinical signs (for example, cough, fever and weight loss), AFS, culture or PCR for *Mtb*, and chest radiography (Table S1 contains a full clinical description of each subject).

### Total TNF-α level cannot distinguish active TB disease from LTBI or non-TB infection

**Figure 1:**
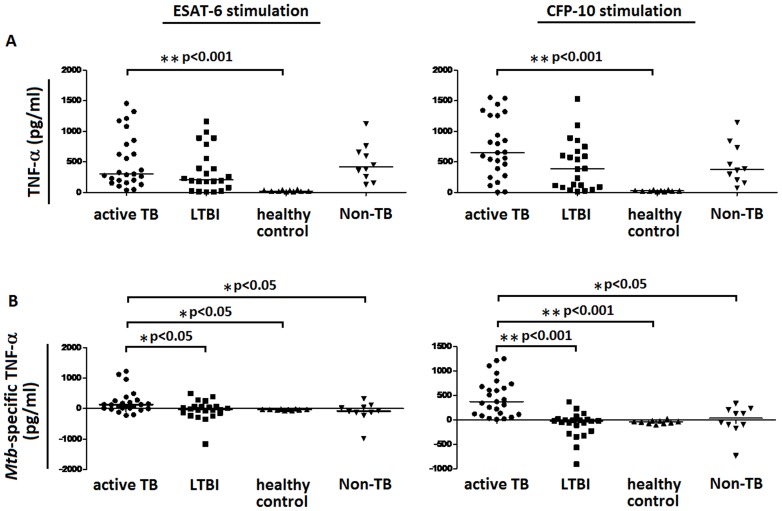
The secretions of TNF-α and *Mtb*-specific TNF-α by ESAT-6 or CFP-10-stimulated PBMCs. PBMCs obtained from active TB patients (n = 25), LTBI individuals (n = 22), healthy control subjects (n = 10) and non-TB patients (n = 10) were stimulated with ESAT-6 or CFP-10. PBMCs stimulated with medium alone were used as a background control. (A) After 16–20 h of incubation, the supernatant was collected and tested for concentrations of secreted TNF-α by ELISA. (B) *Mtb*-specific TNF-α was calculated by subtracting background TNF-α secreted by medium-stimulated PBMCs from TNF-α secreted by ESAT-6 or CFP-10-stimulated PBMCs. Median values for each group of participants are represented by a horizontal bar. *p < 0.05, **p < 0.001.

The secretion of TNF-α by ESAT-6 or CFP-10-stimulated PBMCs from different groups of participants was detected in this study. After stimulation with ESAT-6, the secretion of TNF-α was significantly increased by PBMCs of active TB patients in comparison to healthy volunteer subjects. However, TNF-α secretion had no statistical difference among active TB patients, LTBI individuals, and non-TB patients. Similar results were obtained in CFP-10 stimulation experiment. TNF-α secretion was also significantly increased by PBMCs of active TB patients, as compared with healthy control subjects, and still had no statistical difference as compared with LTBI individuals or non-TB patients (Fig. 1A). These data suggest that although the secretion of TNF-α by ESAT-6 or CFP-10-stimulated PBMCs is significantly increased in active TB group, TNF-α levels do not distinguish active TB from LTBI or non-TB control. Thus, the secretion of TNF-α is not a good biomarker for the diagnosis of active TB disease.

### *Mtb*-specific TNF-α is a potential biomarker for the diagnosis of active TB disease

Given that high background secretion of TNF-α was found in ESAT-6 or CFP-10-stimulated PBMCs from both TB and non-TB patients, we further proposed a new calculation method: *Mtb*-specific TNF-α. *Mtb*-specific TNF-α was defined by the following rule: *Mtb*-specific TNF-α =  TNF-α_ESAT-6 or CFP-10 stimulation_ - TNF-α_background_. Interestingly, after stimulation with ESAT-6 or CFP-10, *Mtb*-specific TNF-α was secreted at significantly higher concentration by PBMCs of active TB patients, as compared with LTBI individuals, healthy control subjects or non-TB control subjects (Fig. 1B). Mean values and standard error of the mean (SEM) of TNF-α and *Mtb*-specific TNF-α secreted by PBMCs of four groups of participants were shown in Table 1. These data suggest that calculation of *Mtb*-specific TNF-α not only distinguish active TB from LTBI, but also can differentiate between active TB and non-TB. Thus, *Mtb*-specific TNF-α is a potential biomarker for the diagnosis of active TB disease.

**Table 1:** Levels of TNF-α and *Mtb*-specific TNF-α secreted by ESAT-6 or CFP-10-stimulated PBMCs of four groups of participants.

	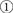 Active TB (n = 25)	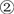 LTBI (n = 22)	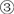 Healthy control (n = 10)	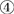 Non-TB control (n = 10)	p 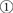 vs 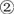	p 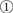 vs 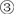	p 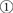 vs 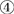
**TNF-α result**							
ESAT-6	511.2±87.40	359.6±76.19	24.61±3.742	487.2±96.68	0.144	<0.001	0.770
CFP-10	733.1±97.74	443.5±87.14	25.17±3.269	464.9±107.3	0.052	<0.001	0.116
***Mtb*** **-specific TNF-α result**							
ESAT-6	209.4±74.80	–57.27±69.26	–39.62±6.555	–114.8±108.0	0.010	0.002	0.014
CFP-10	465.3±76.75	–97.20±57.26	–41.70±10.89	–11.83±96.01	<0.001	<0.001	0.002

Note. Data are expressed as means±SEM; TB: tuberculosis; LTBI: latent tuberculosis infection.

### *Mtb*-specific TNF-α is a prominent biomarker in differentiating between active TB and LTBI

**Figure 2:**
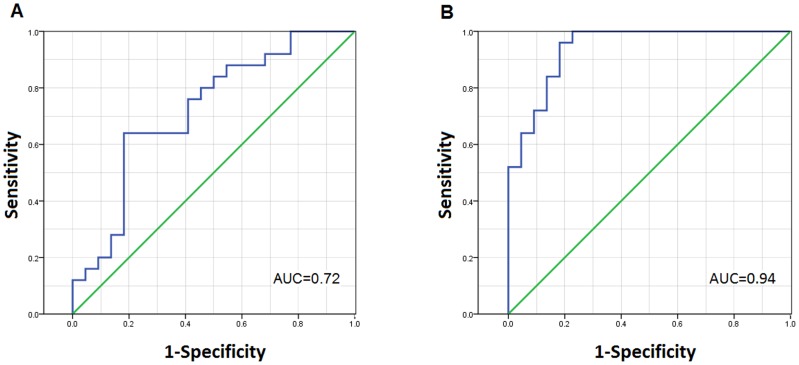
ROC for *Mtb*-specific TNF-α as a classifier to distinguish between active TB and LTBI. With ESAT-6 (A) or CFP-10 (B) stimulation, *Mtb*-specific TNF-α was calculated, then ROC analysis was performed for *Mtb*-specific TNF-α to determine cutoff levels in discriminating between active TB (n = 25) and LTBI (n = 22). AUC  =  Area under the curve.

To differentiate between active TB and LTBI, ROC analysis was then performed to determine the exact cutoff level for *Mtb*-specific TNF-α (Fig. 2). Statistical data of ROC curve for *Mtb*-specific TNF-α were shown in Table 2, using the cutoff value of 71.1 pg/ml for *Mtb*-specific TNF-α in ESAT-6 stimulation experiment, we were able to differentiate active TB from LTBI. Sensitivity and specificity were 64% and 81.82%, respectively. Furthermore, better results were obtained with CFP-10 stimulation. ROC analysis demonstrated sensitivity of 72% and specificity of 90.91%, in discriminating between active TB and LTBI, if using a cutoff value of 136.9 pg/ml. These data suggest that *Mtb*-specific TNF-α is a prominent biomarker in differentiating between active TB and LTBI.

**Table 2:** Statistical analysis of ROC curve for *Mtb*-specific TNF-α to distinguish between active TB and LTBI.

	AUC (95% CI)	P-value	Cut-off	Likelihood ratio	Sensitivity% (95% CI)	Specificity% (95% CI)
**ESAT-6 stimulation**	0.72 (0.57–0.86)	0.009	>71.10	3.52	64 (42.52–82.03)	81.82 (59.72–94.81)
**CFP-10 stimulation**	0.94 (0.87–1.00)	<0.0001	>136.9	7.92	72 (50.61–87.93)	90.91 (70.84–98.88)

Note. TB: tuberculosis; LTBI: latent tuberculosis infection; AUC: area under the curve.

### *Mtb*-specific antigen shows obvious cytotoxicity to PHA-stimulated PBMCs of non-TB patients

**Figure 3:**
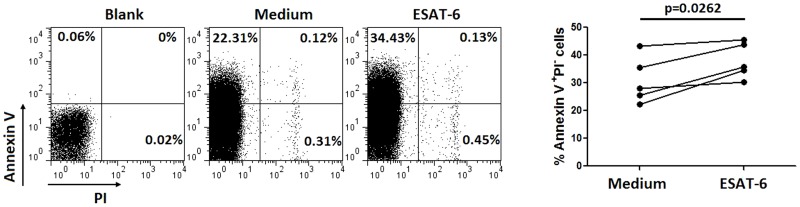
Annexin V staining of medium and ESAT-6-stimulated PBMCs. PBMCs isolated from five non-TB patients were stimulated with 30 µg/ml PHA for 24 h in the presence of 50 µl of ESAT-6 or medium. After culture, PBMCs were collected and the percentages of apoptotic cells (Annexin V^+^PI^−^) were analyzed by flow cytometry. Shown are representative flow cytometry analyses of the apoptotic cells in non-TB patients.

We confirmed in this study that high background secretion of TNF-α, even without any stimulation, was found in PBMCs of both LTBI individuals and non-TB patients. According to this, large negative values of *Mtb*-specific TNF-α which was calculated by subtracting background TNF-α was obtained. We thus investigated why TNF-α secretion by ESAT-6-stimulated PBMCs of non-TB patients was mostly lower than background secretion by these cells. We found that the secretion of TNF-α by PHA-stimulated PBMCs was obvious decreased when adding ESAT-6 to the culture medium (Table 3). This was observed in three non-TB patients. To further determine ESAT-6-induced cytotoxicity, we used flow cytometric analysis with annexin V staining. As shown in Figure 3, the percentage of Annexin V^+^PI^−^ apoptotic cells increased significantly after adding ESAT-6 to the culture medium. These data suggest that *Mtb*-specific antigen like ESAT-6 has obvious cytotoxicity to PHA-stimulated PBMCs of non-TB patients.

**Table 3:** The cytotoxicity of ESAT-6 to PHA-stimulated PBMCs of three non-TB patients.

	non-TB patient 1	non-TB patient 2	non-TB patient 3
clinical signs	fever, cough, headache	fever, plerual effusion	fever, cough, headache
T-SPOT.TB	-	-	**+**
mycobacterial culture	-	-	-
AFS	-	-	-
PCR	-	-	-
**TNF-α secretion by PBMCs (pg/ml)**			
10 µg/ml PHA + 50 µl medium	180±15.5	48.8±5.5	135±13.2
10 µg/ml PHA + 50 µl ESAT-6	95.9±11.2	14.1±3.5	31.8±5.9
20 µg/ml PHA + 50 µl medium	810±34.5	204.1±15.6	650±31.8
20 µg/ml PHA + 50 µl ESAT-6	181.2±19.9	26.5±4.2	145.3±14.5
30 µg/ml PHA + 50 µl medium	1317.6±56.7	300.6±28.4	1123±45.7
30 µg/ml PHA + 50 µl ESAT-6	374.1±23.6	61.8±10.9	305.9±21.4

Note. PHA: phytohemagglutinin; TB: tuberculosis; AFS: acid fast staining.

## Discussion

Commercial IGRAs such as the ELISA-based Quantiferon TB Gold In-Tube assay (QFT-IT) and the ELISPOT-based T-SPOT.TB, are widely used especially in high income, low burden settings for the diagnosis of *Mtb* infection. These assays have proven to be useful to accurately detect *Mtb* infection [13], ; however, they cannot reliably discriminate between active TB and LTBI [15]. Previous studies have shown that multi-cytokine analysis after stimulation of PBMCs from patients with latent or active TB infection indicates the possibility of successfully discerning these two disease states [16], [17]. However, no biomarker is currently ready for routine clinical use in distinguishing active TB from LTBI.

TNF-α is a cytokine with a long history in TB research and plays important roles in immune and pathological responses of TB patients [18]. Kupeli et al. showed that bronchoalveolar lavage fluid TNF-α level is found to be significantly increased in TB patients compared with both other pulmonary disease patients and healthy controls [19]. Pleural fluid TNF-α level also proved to be useful in prognosticating tuberculous pleurisy with high sensitivity and specificity rates [20]. Recently, Harari et al. proposed that the proportion of single-positive TNF-α *Mtb*-specific CD4^+^ T cells might be used as a signature of active TB disease [21]. Until now, however, the soluble *Mtb*-specific TNF-α secretion has not been fully evaluated in TB and non-TB patients.

In this study, we used *Mtb*-specific antigens such as ESAT-6 and CFP-10 to stimulate PBMCs isolated from active TB patients, LTBI individuals, non-TB patients and healthy volunteer subjects. We found that TNF-α secretion is not a good biomarker for the diagnosis of active TB disease because it cannot distinguish active TB from LTBI or non-TB. Interestingly, we found that although TNF-α secretion by PBMCs of LTBI individuals and non-TB patients was increased, the background secretion of TNF-α was also very high in these groups. Previous studies have shown that background level of TNF-α is observed in un-stimulated samples from patients whether infected with *Mtb* or not [22], [23]. We confirmed in this study that high background secretion of TNF-α was found in PBMCs of non-TB patients. We also investigated why TNF-α secretion by ESAT-6-stimulated PBMCs of non-TB patients was mostly lower than background secretion by these cells. We found that *Mtb*-specific antigen like ESAT-6 has obvious cytotoxicity to PHA-stimulated PBMCs of non-TB patients.

According to high level of background TNF-α secreted by PBMCs of LTBI individuals and non-TB patients, we proposed *Mtb*-specific TNF-α which is calculated by subtracting background level of TNF-α secreted by unstimulated PBMCs. Excitingly, we found that calculation of *Mtb*-specific TNF-α was substantially better than direct detection of TNF-α for the diagnosis of active TB disease.

Taken together, these data indicate that *Mtb*-specific TNF-α secreted by ESAT-6 or CFP-10-stimulated PBMCs is a new biomarker in distinguishing active TB from LTBI or non-TB. However, the sample size in this study was small, which may lead to inaccurate sensitivity and specificity. Moreover, the diagnosis of active TB was difficult sometimes. Some active TB patients may have no obvious clinical or radiographic evidence, leading to the wrong diagnosis and causing unmatched results. Thus even if intriguing, our findings should be confirmed in larger TB populations.

## Supporting Information

Table S1 **Clinical description of patients diagnosed with active TB disease.**
(TIF)
