# Supplementary figures and images for: Mycobacterium Tuberculosis-Specific TNF-α Is a Potential Biomarker for the Rapid Diagnosis of Active Tuberculosis Disease in Chinese Population
Source: PLoS One. 2013 Nov 11;8(11):e79431. doi: 10.1371/journal.pone.0079431 (PMC3823617; doi:10.1371/journal.pone.0079431)

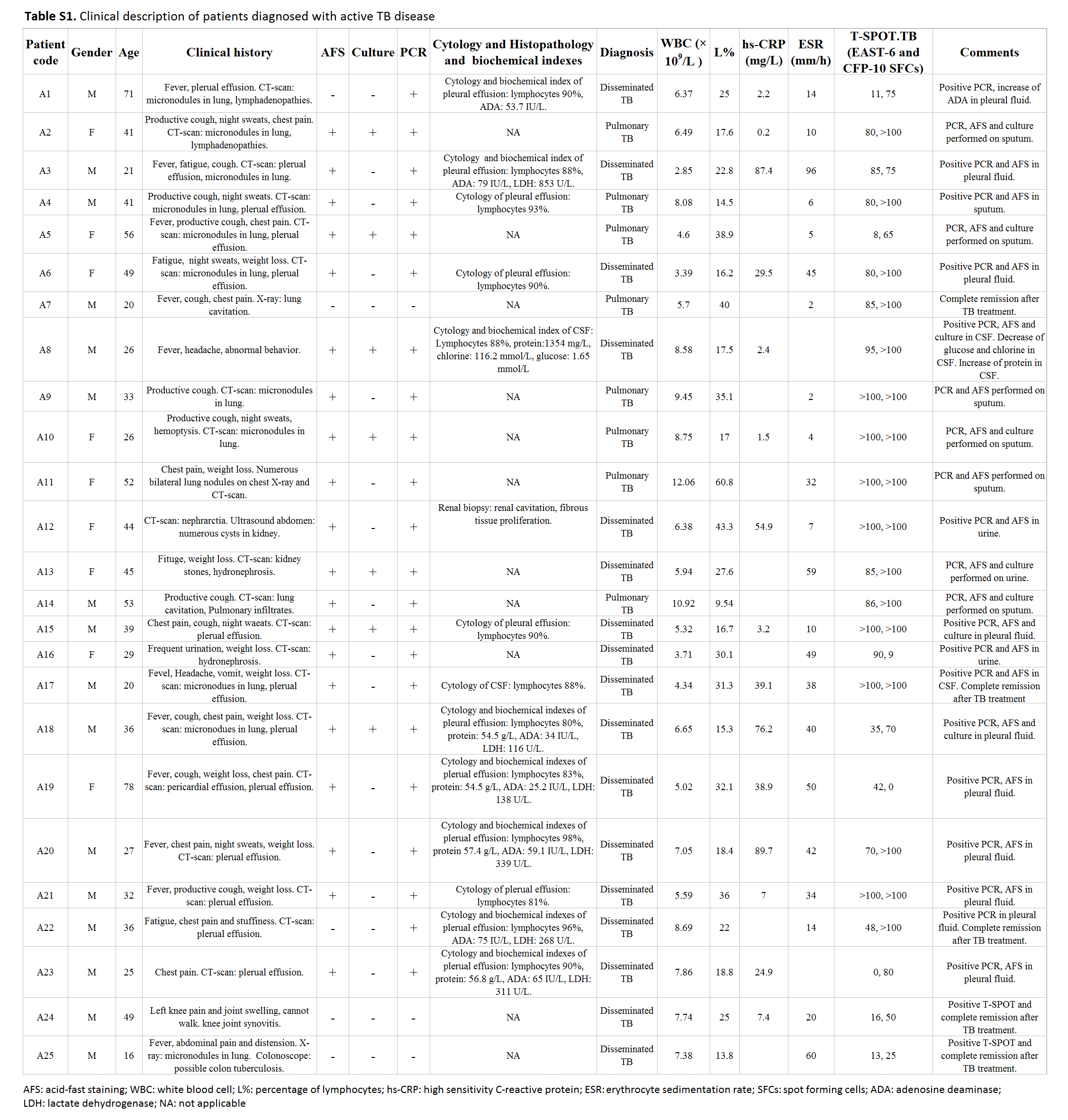

Supplement: Table S1 — Clinical description of patients diagnosed with active TB disease. (TIF) [file pone.0079431.s001.tif]
